# Novel Materials for Sustainable Energy Conversion and Storage

**DOI:** 10.3390/ma13112475

**Published:** 2020-05-29

**Authors:** Jung Kyu Kim

**Affiliations:** School of Chemical Engineering, Sungkyunkwan University (SKKU), Suwon 16419, Korea; legkim@skku.edu; Tel.: +82-31-290-7254

**Keywords:** device architecture, energy storage battery, solar cells, electrocatalysis, photoelectrochemical cells, solar fuels

## Abstract

Sustainability is highly desired for human beings due to a rapidly changing global climate and numerous environmental issues. In past decades, state-of-the-art studies have been extensively conducted to achieve sustainable energy conversion and storage. However, the remaining challenges in the commercialization of energy conversion and storage devices are to develop novel materials and advanced manufacturing processes. Furthermore, the engineering of nanostructures and device-architectures is of great importance for the energy conversion and storage flat forms. This Special Issue “Novel Materials for Sustainable Energy Conversion and Storage” aims the state-of-the-art research reports of novel nanomaterials and the engineering of device architectures for divergent energy conversion and storage applications with high sustainability involving solar energy systems, electrochemical cells, artificial photosynthesis or secondary (rechargeable) batteries, as highlighted in this editorial.

The study of energy conversion and storage is an essential research field for the sustainable development of humanity, which has attracted the significant attention of worldwide researchers in recent decades. For energy conversion, the conversion of solar energy into another type of energy we can easily use has been spotlighted due to the accelerated crucial climate changes and the increasing demand of renewable energy. From the perspective of energy conversions from solar energy, photovoltaics and photoelectrochemical (PEC) cells can be good energy conversions converting solar energy into electric or chemical energies, respectively [1,2]. As a solar-to-electric energy conversion device, the photovoltaic has been extensively studied for several decades, of which the power conversion efficiency (PCE) is determined by the number of photogenerated charge carriers and their efficient collection [3]. Especially in the case of polymer-based bulk-heterojunction (BHJ) solar cells, it is highly required to adjust the valance of photoinduced electrons and holes for the high PCE due to the short exciton diffusion length in BHJ [3]. To achieve high efficiency, PEC cells in recent studies have been subject to the enhancement of three components including light harvesting (or absorptance), charge separation (or charge transport) and charge transfer (or kinetics) efficiencies, strongly associated with the solar to hydrogen conversion efficiency via solar water splitting [4]. In addition, the electrochemical water splitting involving the oxygen evolution and hydrogen reduction reactions facilitates the energy conversion from electricity into chemical energy [2]. Considering the high-energy density and three important factors for substituting fossil fuels (i.e., the generation, conversion and storage), hydrogen can be considered an ideal energy carrier, which can replace the widely used fossil fuels such as natural gas or gasoline. Thus, the energy conversion to produce hydrogen fuels can be a promising strategy for the storage of chemical energy. For the electric energy storage, the commercialized Li-ion batteries (LiBs) have been widely utilized in various electronics and vehicles in recent years due to the high-energy density and cost-effectiveness of their maintenance. However, the increasing price of Li metal and their safety issues are recognized as critical drawbacks of LiBs [5]. In recent years, Na-ion batteries (SiBs) have been extensively studied to replace the LiBs in the field of large-scale energy storage systems due to their earthly abundance and the low cost of sodium [5]. However, compared to LiBs, SiBs suffer from the lower capacity and poor cycling stability due to the much larger ionic radius of the Na ion than that of the Li ion. Thus, the development of novel materials with tailored nanostructures and modified physicochemical properties is an indispensable part of energy conversion and storage applications.

This Special Issue “Novel Materials for Sustainable Energy Conversion and Storage” has been proposed to present the state-of-the-art studies on novel nanomaterials and the engineering of device architectures for divergent energy conversion and storage applications with high sustainability. For this reason, the seven articles, namely the two reviews and five research articles that constitute this Special Issue, investigate different aspects of sustainable energy conversion and storage, of which a brief summary is provided in the following. 

Thanks to the precious contributions of Professor Uk Sim, two review articles were published, of which the subject was novel materials for water splitting via photoelectrochemical or electrochemical reactions. Kim et al. [6] recently reviewed the reported solar water splitting via PEC cells comprised of metal–organic halide perovskite-based electrodes. In their review, the challenges remained in perovskite-based solar fuel production including the conversion efficiency and the stability properties were discussed. Meanwhile, Lim et al. [7] reviewed the water-splitting catalysts composed of the low dimensional (LD) carbon-based novel materials. The significant catalytic activity of LD carbon catalysts with unique electrical, mechanical and catalytic properties were summarized. 

Among the research articles, the subject of two articles was photovoltaics, for another two the subject was water splitting and the other one was Na/Li storage. As for the photovoltaics, Gaspar et al. [8] studied a bulk-heterojunction (BHJ) polymer solar cell comprised of functionalized fullerene side chain with thiophene and carbazole moieties. They demonstrated that the molecular structure of acceptor material can play an important role for enhancing device performance. Xu et al. [9] proposed an artificial neural network (ANN) to predict the current to potential curves of a silicon-based photovoltaic module at an arbitrary irradiance and temperature. 

For the water splitting, Woo et al. [10] reported highly efficient electrocatalysts for overall water splitting to produce hydrogen by using a facile electrospinning technique. For the efficient hydrogen evolution reaction (HER) and oxygen evolution reaction (OER), Co–CeO_2_ and Ni_2_Fe nanoparticles were incorporated into the carbon nanofibers (CNFs), respectively. As a result, a remarkably lower overpotential and a long-term durability over 70 h of overall water-splitting reaction were observed by using the Co–CeO_2_@CNF and Ni_2_Fe@CNF electrodes. Alam et al. [11] harnessed reduced graphene oxide (rGO) catalysts for enhancing PEC HER. In their report, the rGO prepared by a plasma-enhanced chemical vapor deposition (PECVD) method was deposited on a p-type Si wafer to fabricate a photocathode, of which the PEC HER performance was outstandingly higher than hydrophilic graphene oxide-based photocathode.

In the field of energy storage, Liu et al. [12] used a one-pot hydrothermal method to fabricate nanoparticulate nickel selenide (NiSe)-decorated rGO nanosheets (NiSe/rGO). The NiSe/rGO nanocomposites were utilized as an anode material for SIBs and LIBs. The synergic effects between NiSe and rGO induced the high conductivity and large specific surface area, which was attributed to the enhanced reversible specific capacity and electrochemical performance for Na/Li storage.
